# First-Arrival Differential Counting for SPAD Array Design †

**DOI:** 10.3390/s23239445

**Published:** 2023-11-27

**Authors:** Mel White, Tianyi Zhang, Akshat Dave, Shahaboddin Ghajari, Alyosha Molnar, Ashok Veeraraghavan

**Affiliations:** 1 Department of Electrical and Computer Engineering, Rice University, Houston, TX 77005, USA; tz12@rice.edu (T.Z.); vashok@rice.edu (A.V.); 2 Media Laboratory, Massachusetts Institute of Technology, Cambridge, MA 02139, USA; ad74@mit.edu; 3 Department of Electrical and Computer Engineering, Cornell University, Ithaca, NY 14853, USA; sg2367@cornell.edu (S.G.); am699@cornell.edu (A.M.)

**Keywords:** SPAD, LiDAR, computational imaging, compressed sensing, photon counting, HDR imaging

## Abstract

We present a novel architecture for the design of single-photon detecting arrays that captures relative intensity or timing information from a scene, rather than absolute. The proposed method for capturing relative information between pixels or groups of pixels requires very little circuitry, and thus allows for a significantly higher pixel packing factor than is possible with per-pixel TDC approaches. The inherently compressive nature of the differential measurements also reduces data throughput and lends itself to physical implementations of compressed sensing, such as Haar wavelets. We demonstrate this technique for HDR imaging and LiDAR, and describe possible future applications.

## 1. Introduction

SPADs (Single-Photon Avalanche Diodes) are capable of sensing individual photons, and thus are utilized heavily when the application is photon-starved, such as in biomedical imaging [1], or for 3D range-finding, where the exact time of travel of a photon can be used to calculate depth [2]. Many SPAD cameras contain only a single pixel which is scanned across a scene, but it is often preferable to have a full array of SPAD pixels. SPAD arrays typically have low fill factor, however, owing to the complex circuitry required for collecting photon timestamps, and high data throughput generated by a large number of SPADs.

We present novel readout architecture which addresses both challenges at once: first arrival differential SPADs (FAD-SPADs) [3]. Our hardware consists of small digital circuitry at the pixels that record relative, rather than absolute, information about flux or time of flight. This technique differs from previously proposed hardware solutions such as TDC sharing, adaptive sensing [4], data sketching [5], and sensor fusion [6,7] because it requires either no TDCs or few TDCs (depending on the application; see Table 1). Our key insight is that rich information is encoded in just the first photon captured within a time window; we do not necessarily require precise time stamps or fine-grained histograms of photon arrival information for many tasks. Moreover, this information can be recorded with minimal support circuitry. Thus, our method can also provide gains in certain imaging metrics, including significantly reduced circuit footprint and better pixel packing, orders of magnitude data size reduction, and improved dynamic range.

In this paper, we build upon the results presented at IISW 2023 [3] and extend our analyses of the design parameters. We demonstrate two applications for the use of FAD-SPADs, summarized in Table 1: first, a differential flux sensing application that results in high dynamic range images, and second, a relative photon timing application for depth sensing that allows us to implement high-resolution LiDAR (light detection and ranging) with significantly reduced data throughput. We discuss challenges specific to this design and methods to overcome them, and consider the outlook and possible future applications of our work.

## 2. Prior Work: SPAD Arrays

SPADs function by reverse-biasing a diode past its breakdown voltage, such that the energy of a single incident photon is enough to set off an “avalanche” of charge. This charge avalanche is read out as a pulse (for example, by triggering a digital buffer), and the SPAD is reset to its initial state, either by independent asynchronous quenching circuits, or a global reset. We are interested in either the number of pulses on a SPAD pixel, or the exact time of arrival of the pulse with respect to the source (e.g., a laser).

To perform photon-counting, a digital counter suffices, and this digital counter may be positioned outside of the array. To extract the time of arrival, on the other hand, requires a time-to-digital converter (TDC), which may be several times larger than the SPAD itself, and must be placed immediately next to the SPAD for accuracy. A high-resolution TDC per pixel is idea for most applications, but the large area occupied by the TDC reduces the fill factor (area of the array that is occupied by active SPADs divided by the total array area); it is common to see a fill factor of 1–10% in per-pixel TDC arrays [8,9,10,11,12,13,14]. In addition, a higher bit depth of timing information increases the bandwidth, which in turn reduces the frame rate. For a fuller discussion of these trade-offs, see [15,16].

To address these challenges, some alternatives to a per-pixel TDC architecture have been proposed [17]. TDC sharing is a common approach, wherein one TDC processes arrival times for multiple SPADs. The group of SPADs may operate as a single super-pixel, as in [18,19,20] or as individual pixels, as in [4,21,22,23]. In the first case, the effective number of pixels is reduced, but fill factor is still improved. The latter requires additional circuitry to report the sub-location within the group of SPADs that recorded the photon event, and so while it preserves the number of pixels, there is a cost to the fill factor and complexity of readout.

Another alternative is time-gating, which requires precisely controlling the time during which the SPAD is active, and counting the photons that occur within that window, as in [24,25,26,27,28,29]. Since the counting mechanism doesn’t require a TDC, the pixels can be spaced more closely, allowing many more pixels per array (recently, up to 1 megapixel [30]). However, good timing precision requires sequentially scanning many short time windows, which slows readout. Histogram-based photon counting methods can also be implemented with TDCs, eliminating the need to scan many windows, but this sacrifices spatial resolution.

To overcome some of the previously described tradeoffs, 3D stacking fabrication has been proposed, wherein the active devices are fabricated on one wafer, and the supporting electronics are fabricated on another wafer, which are then sandwiched together. Since the supporting electronics are beneath the sensors, they do not impose a cost to the fill factor. Per-pixel TDCs [31,32], shared TDC architectures [33,34,35,36], and time gating [37] have been implemented with this approach. It also allows the designer to choose separate processes for the SPAD and supporting electronics that are optimal for each. The trade-off for this approach is in manufacturing complexity and cost; the wafers must be perfectly aligned, and additional delays or parasitics between the wafers must be accounted for. This approach also typically requires back-side illumination, which in turn requires careful thinning of the SPAD wafer substrate.

Recently, there have also been some unconventional architectures proposed. Severini et al. [38] demonstrate a TDC-free architecture that employs an event-driven logic for photon coincidence detection. Others have proposed theoretical hardware yet to be built. Sheehan et al. [5] describe a “sketching” framework that reports statistical information about the histogram, thus significantly reducing the bandwidth. This approach has would require the use of TDCs as well as significant additional support circuitry on-chip for the required calculations. Ingle and Maier [39] propose a histogramming approach that does not require TDCs or counters; instead, lightweight digital circuitry adjusts the width of histogram bins, and these bin boundaries are the reported information.

**Figure 1:**
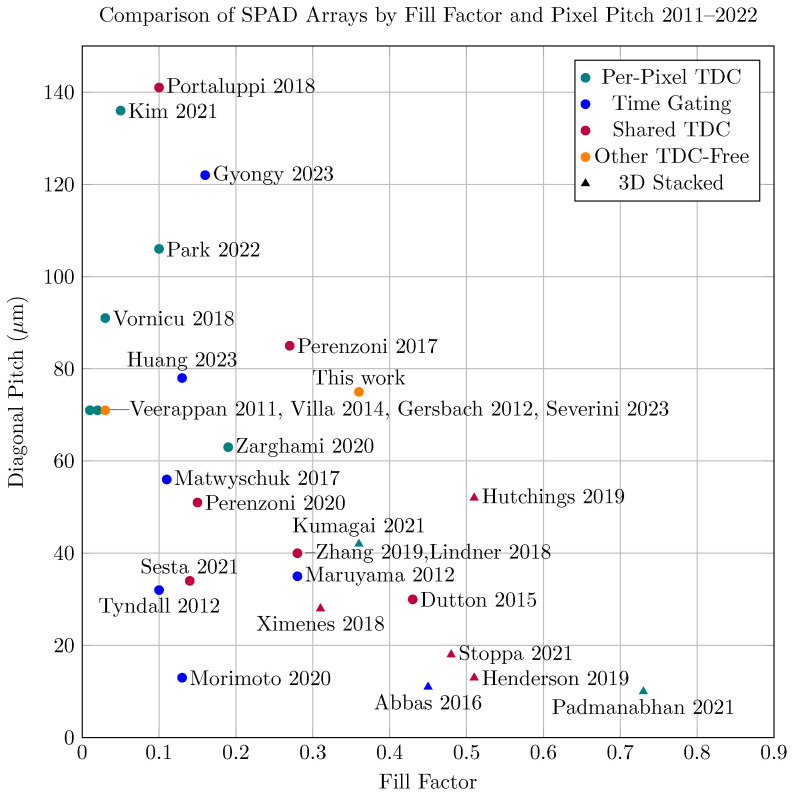
A comparison of SPAD array fill and pixel pitch for arrays employing different techniques. The diagonal pitch was used to normalize the comparison between isotropic and anisotropic pixels. Circular markers indicate a single-wafer approach, and triangular markers indicate 3D stacking, while colors organize the work into architecture types: Per-Pixel TDC [8,9,10,11,12,13,14], Shared TDC [4,18,19,20,21,22,23], Time-Gating [24,25,26,27,28,29], and TDC-Free [38,40]. The fill and pitch were not given explicitly for [11,12,29,31,32], and were estimated based on information provided. The values provided for our method are based on a prototype in 180 nm CMOS.

A comparison of the physically-implemented arrays of the last decade by pixel pitch and fill factor is given in Figure 1. While the overall number of pixels, CMOS process, and other considerations that affect array design are not explicitly shown here, there are some clear trends; per-pixel TDC approaches fare the worst in terms of fill factor and spacing, time-gated and shared-TDC methods do better, and 3D stacking is the clear winner. Of the single-wafer methods, ours achieves better fill than all but one [25].

## 3. Key Concept: The FAD Unit

**Figure 2:**
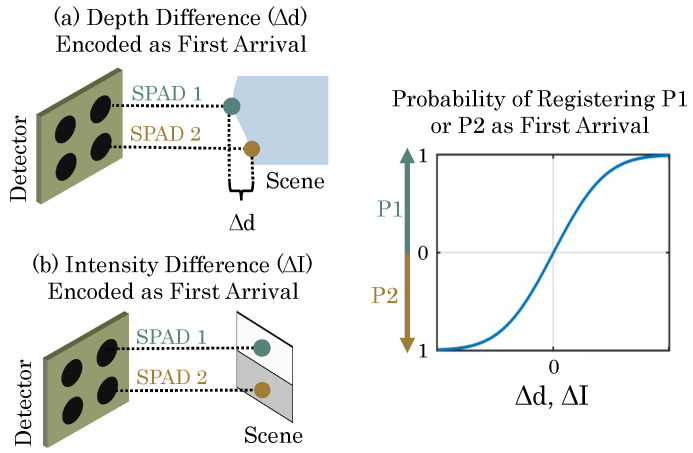
FAD-SPAD operation principle. 1: Either depth intensity differences can be encoded with the first arrival of a photon within a time window. 2: The relationship between the first arrival and the probability of recording an up or down count is nonlinear and monotonic. This figure is a modified reproduction, used with permission from [41].

Consider two SPADs, S1 and S2, and a time window, T. In an active-lighting scenario, where a laser is pulsed at the scene, whichever SPAD pixel is focused at a nearer point in the scene is more likely to receive a photon first within T. Likewise, in passive lighting, whichever SPAD sees a higher flux is more likely to see a photon first within T. Over a large number of time windows, the probability of a first arrival on S1 versus S2 is a nonlinear, but monotonic function of the depth or intensity difference. By recording the total number of first arrivals on each SPAD, we can estimate depth or flux difference. This principle is illustrated in Figure 2.

### 3.1. Implementation

**Figure 3:**
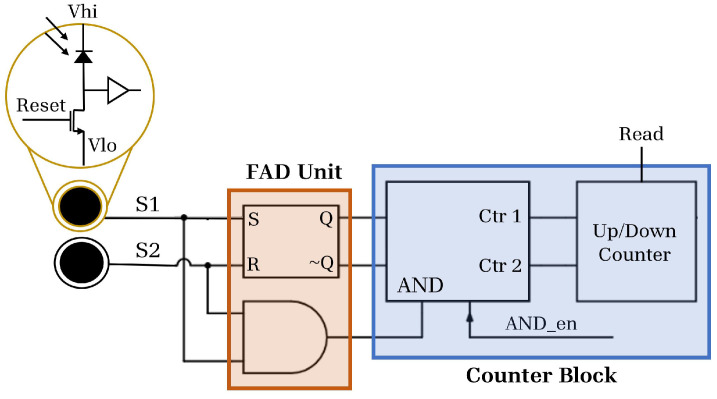
FAD-SPAD readout circuitry. Here, we show two SPADs with global, active reset as the inputs to an SR latch. This is a modified version of a figure used by permission from [40].

To obtain the first arrival measurement, we use an SR (set-reset) latch, which locks in the first logical high signal to arrive at its inputs, and holds that value until it is reset. A SPAD is connected to each input, so that the first photon arrival within a window excites a SPAD response, and that signal is locked in at the SR latch. The output of this SR latch is connected to an up/down counter (with some control logic). If SPAD 1 saw the first arrival, the counter will increment by one; conversely, if the first arrival was at SPAD 2, the counter will decrement by one. A simplified diagram is given in Figure 3.

Crucially, the only circuitry that need be placed within the pixel structure is the FAD unit; the counter and its associated logic can be placed at the edge of the array. Ideally, the nets connecting SPADs to the SR latch should be path-length matched and as short as possible to avoid race conditions or biases introduced by delays or parasitics.

In a typical frame, a time window T for the SPADs is set, as well as a number of subframes N. One subframe consists of resetting all of the SPADs and SR latches, and allowing the SPADs to be active for time T. Once first arrival information is locked in by the FAD units, the latch state for each latch is passed off-array to the counters. This process is repeated N times, after which the final state of the counters is read out. The next frame can begin accumulating during the previous frame’s readout phase, so that the frame rate is limited only by the user-defined T and N.

#### 3.1.1. Distinguishing between Single and Dual Photon Events

**Figure 4:**
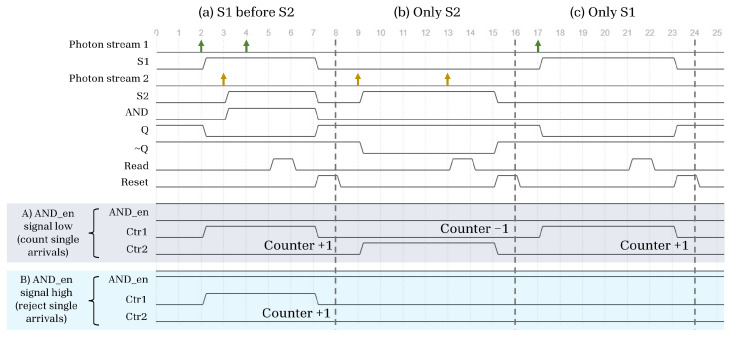
Example timing diagram illustrating possible arrival sequences from the photon streams at two SPADs, S1 and S2, and the associated counter increments under two different modes of operation, where the AND gate is either enabled or disabled.

In some cases, we may be interested in recording up/down counts only during windows where there were dual arrivals (that is, both SPAD 1 and SPAD 2 registered a photon arrival). To account for this, we also include an AND gate in parallel with the SR latch, the output of which is optionally connected to the counter control. When enabled, only time windows with dual arrival events will contribute to increments or decrements on the counter. Some example photon arrival sequences and their associated timing diagrams for the circuit in Figure 3 are given in Figure 4.

#### 3.1.2. Recording the Sum Total Photon Events

Additionally, the total number of windows where any photon was detected can be counted by connecting the SPAD outputs to an XOR tree. In this way, we can obtain a sum or difference of first-arrival events between any two arbitrary groups of pixels.

### 3.2. FAD Connections

**Figure 5:**
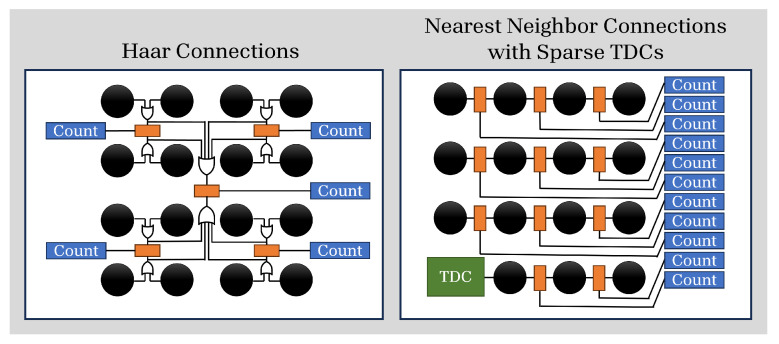
Two possible ways of connecting SPAD outputs via FAD units (illustrated as orange blocks) and counters (blue blocks). Layered connections, such as a Haar wavelet configuration, can be achieved by OR-ing outputs of SPADs together in selected groups prior to the input of the FAD unit. Alternatively, the designer could place FAD units between neighboring pixels, as shown on the right.

Multiple SPADs can also be connected to one of the two inputs of an SR latch, allowing for various configurations using the same core FAD circuit. For example, we may pass the output of SPAD 1 and SPAD 2 to an OR gate, and the outputs of SPAD 3 and SPAD 4 to a separate OR gate, and place a FAD unit between these two groups such that the counter increments if the arrival is at S1 or S2, or decrements if the first arrival is at S3 or S4. Any arbitrary number of such groups can be built, and the groups may overlap (e.g., S1|S2 vs. S3|S4 and S1|S3 vs. S2|S4). Overlapping groups will record differential measurements simultaneously. This is illustrated in Figure 5 in the left panel, and contrasted with the nearest neighbors connectivity scheme in the right panel. This figure shows only some of the connections in each configuration as an illustrative example. The left panel example, Haar groupings, shows how two layers of vertical differential measurements can be made with OR gates. This layout is selected for our example of intensity imaging with passive lighting (Section 4.1). The right panel shows an example of connections with nearest neighbors, with no heirarchical layers of measurements, and a few sparse TDCs. This configuration will be demonstrated for flash LiDAR in Section 5.

## 4. The Passive Regime: Encoding the First Arrival as Intensity Difference

SPADs are ideal for extreme low-light conditions, where their single-photon sensitivity allows detection of extremely small light flux. However, they saturate quickly when the flux exceeds one photon per window. When a SPAD experiences an avalanche, any subsequent photon arrivals are lost; this is referred to as pile-up. Given the pile-up issue at relatively low fluxes, it may be surprising that these devices can be used for high dynamic range imaging. Yet, numerous researchers have exploited the statistics of photon arrivals to obtain high dynamic range information [42,43,44]. Alternatively, one can use multiple exposures with single-photon sensors, as is done for standard CMOS [45]. Our approach differs from prior work on HDR imaging in that it is done with a single exposure, and no TDCs. The following section builds on work that was published with the name “D-SPAD” [40] (Differential SPAD) instead of “FAD-SPAD” (First-Arrival Differential SPAD). The principle is identical; only the name was updated to emphasize that the first arrival encodes the differential information to high-dynamic range intensity imaging.

### 4.1. Choice of Architecture

In this case, we will be interested in very low flux conditions that produce only single-arrival events, as well high flux conditions that produce dual-arrival events, so we will not enable the AND functionality described in Section 3.1; we will not limit which events can impact the counter.

**Figure 6:**
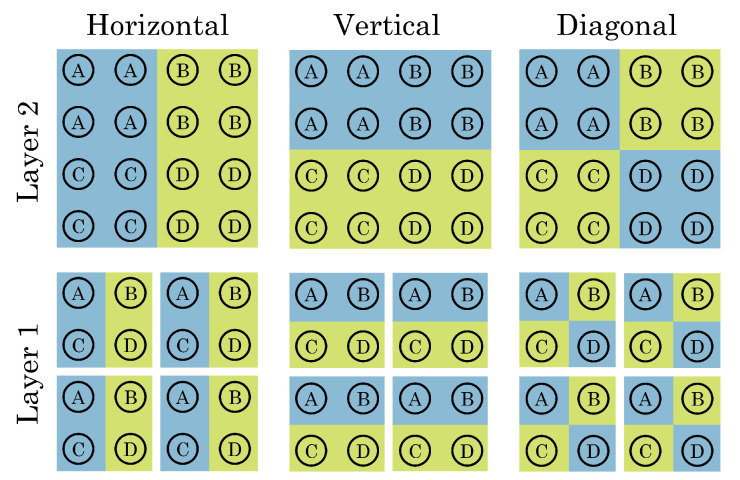
An illustration of Haar groupings for a 4 × 4 array. If the first photon in a window hits a blue-shaded pixel, the counter counts up, and if it hits a green-shaded pixel, it counts down. Horizontal measurements are taken by grouping A and C pixels against B and D pixels, vertical measurements are taken by grouping A and B pixels against C and D pixels, and diagonal measurements are taken by grouping A and D pixels against B and C pixels. At layer 1, each SPAD “group” consists of one pixel (operating as A, B, C, or D). At layer 2, four SPADs operate as a single SPAD by OR-ing their outputs together. Additional layers would have have 16 SPADs per label. Note that all of these connections are simultaneous, such that if the first photon arrival occurs at the bottom right pixel, this will cause up counts on the horizontal and vertical measurements of both layer 1 and 2, and down counts on the diagonal measurements of layer 1 and layer 2. This is a modified version of a figure by [40].

We also are interested in compressing the reported information, and so we will choose a grouped architecture for the FAD connections. A Haar-like grouping structure is a natural choice for image processing, especially where the sensor output is already binary [46]. To do so, we cluster all pixels on the left and right half of the chip together, and link these groups by FAD. We do the same for the top and bottom, and then for diagonal groups. We then divide the array into four sub-groups, and repeat the process for each sub-group, as shown in Figure 6.

### 4.2. Principle and Mathematical Formulation

Take the fluxes at the two SPADs to be $\Phi_{1}$ and $\Phi_{2}$ (photons per cycle), and assume Poisson arrival processes at both SPADs. Take *T* as the time window where the two SPADs are active. If the background flux is very low (≤1 photon/window), the expected FAD-SPAD output E[FAD] will be no different than if there were two independently recorded SPADs:

(1) $$\begin{array}{c} E[FAD] \end{array}=N_{cycles}\left[e^{-\Phi_{1}T}-e^{-\Phi_{2}T}\right]$$

However, under high flux, most windows will have dual arrivals (a response on both SPADs) and thus suffer from pile-up. Under this condition, the probability of a given number of up/down counts is:

(2) $$\begin{array}{c} E[FAD] \end{array}=N_{cycles}\left[1-e^{-(\Phi_{1}+\Phi_{2})T}\right]\left(\frac{\Phi_{1}-\Phi_{2}}{\Phi_{1}+\Phi_{2}}\right)\;$$

where *T* is the period of the detection cycle.

Knowing this relationship, we can estimate the flux from counts by optimizing the following least squares objective:

(3) $$\min_{\Phi }\left(∥{fad_{agg}-E\left[FAD_{agg}|\Phi \right]∥}_{2}^{2}+{∥n_{sum}-E\left[N_{sum}|\Phi \right]∥}_{2}^{2}\right)$$

where $fad_{agg}$ and $FAD_{agg}$ are the measured and estimated aggregate output of the difference operations, and $n_{sum}$ and $N_{sum}$ are the measured and estimated total number of photon arrivals.

### 4.3. Analysis

Equation (2) offers some insight as to why this architecture allows for high dynamic range imaging. The output is scaled in the denominator by the sum of the fluxes, which prevents the counts from saturating at high flux. Two types of saturation can occur:

1. at the counter, where digital bit storage saturates, and
2. at the pixel, where early photon arrivals mask later arrivals.

**Figure 7:**
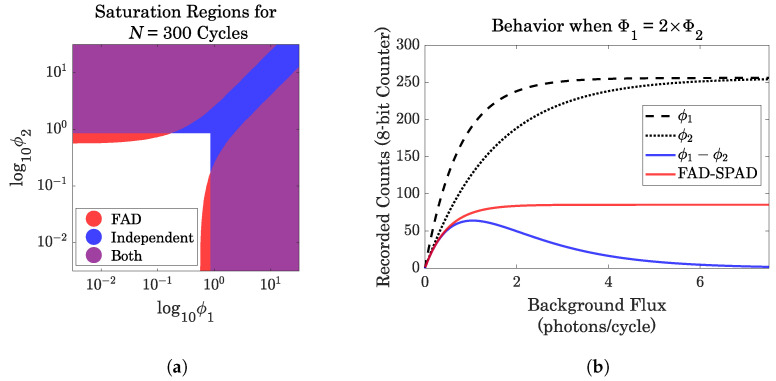
Comparison of saturation in FAD and independent (per-pixel TDC) architectures. (**a**) Regions of saturation; (**b**) Subtraction versus FAD. Reproduced with permission from [40].

In Case 1, the FAD-SPAD architecture allows the recording of differential measurements even when both counters (on SPAD 1 and SPAD 2) would have saturated independently. The background fluxes cancel out, as long as they are similar in magnitude. See Figure 7, left; the region shaded in blue indicates when independently-operating SPADs would saturate their counters, but FAD-SPADs preserve differences. In Case 2, the FAD-SPAD architecture again preserves small differences in flux. In a global-reset configuration, if two neighboring pixels each saw at least one photon during every window, then any small difference in flux between them will be lost. However, since the FAD-SPADs still record which was first, flux differences can be discerned even under pile-up conditions.

#### Dynamic Range Analysis

Typically, we define dynamic range by the largest and smallest signals detectable. Since we are here not measuring a signal directly, we instead consider the dynamic range of the largest and smallest difference in signals that are detectable, which will depend on background flux conditions. We begin by redefining $\Phi_{1},\Phi_{2}$ in terms of background flux $\Phi_{0}$ and a small differential flux $\Phi_{D}$ as $\Phi_{1}=\Phi_{0}+\Phi_{D}$ and $\Phi_{2}=\Phi_{0}-\Phi_{D}$.

Re-writing Equation (2), the probability of a count on differential SPAD in terms of $\Phi_{0}$ is:

(4) $$\hat{p}=\left(1-e^{\left(-2\Phi_{0}\right)}\right)\frac{\Phi_{D}}{\Phi_{0}}$$

Under high flux conditions, the exponential term goes to zero and the variance of the estimated differential SPAD counts is:

(5) $$\begin{array}{c} \sigma_{C} \end{array}=\sqrt{N\frac{\Phi_{D}}{\Phi_{0}}\left(1-\frac{\Phi_{D}}{\Phi_{0}}\right)}$$

The smallest detectable signal is determined by the ratio of the standard deviation to the expected value, $N\hat{p}$:

(6) $$\begin{array}{cc} {\left(\frac{\sigma_{C}}{N\hat{p}}\right)}_{FAD} & =\frac{\sqrt{N\frac{\Phi_{D}}{\Phi_{0}}\left(1-\frac{\Phi_{D}}{\Phi_{0}}\right)}}{N\frac{\Phi_{D}}{\Phi_{0}}}=\frac{\sqrt{1-\frac{\Phi_{D}}{\Phi_{0}}}}{\sqrt{N\frac{\Phi_{D}}{\Phi_{0}}}} \end{array}$$

We can repeat the same analysis for independently operating SPADs:

(7) $${\left(\frac{\sigma_{C}}{N\hat{p}}\right)}_{I-SPAD}=\frac{\sqrt{Ne^{\Phi_{0}\left(1-\frac{\Phi_{D}}{\Phi_{0}}\right)}\left(1-e^{\Phi_{0}\left(1-\frac{\Phi_{D}}{\Phi_{0}}\right)}\right)}}{Ne^{\Phi_{0}\left(1-\frac{\Phi_{D}}{\Phi_{0}}\right)}}=\frac{\sqrt{1-e^{\Phi_{0}\left(1-\frac{\Phi_{D}}{\Phi_{0}}\right)}}}{\sqrt{Ne^{\Phi_{0}\left(1-\frac{\Phi_{D}}{\Phi_{0}}\right)}}}$$

**Figure 8:**
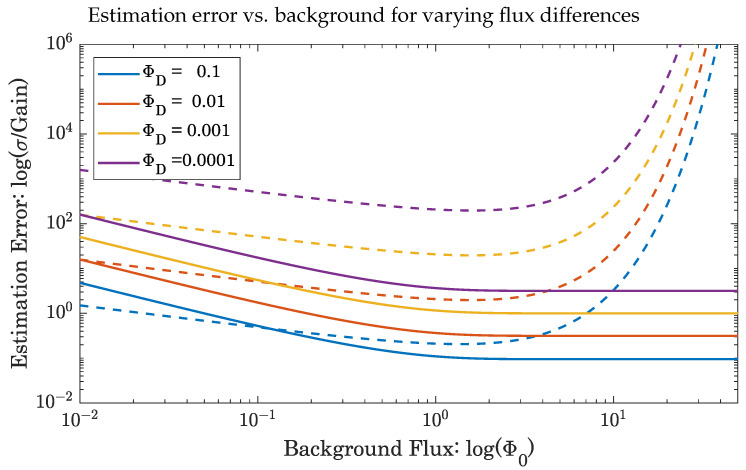
Solid lines represent the FAD-SPAD estimation error, and dotted lines represent independent SPADs. Colors correspond to different differential fluxes. Note that at high background flux, the estimation error for independently operating SPADs goes to infinity, as the background overwhelms small signals. FAD-SPADs, on the other hand, have an estimation error that saturates under high background.

Figure 8 illustrates the behavior of the estimation error for increasing background flux ($\Phi_{0}$), and several flux differences ($\Phi_{D}$). Independently operating SPADs perform similarly to FAD-SPADs under low background flux, but their estimation error increases exponentially as the background flux becomes greater than 1 photon per window. Meanwhile, the estimation error of the differential flux for the FAD-SPAD decreases to its minimum value, regardless of the background.

### 4.4. Simulation Results

**Figure 9:**
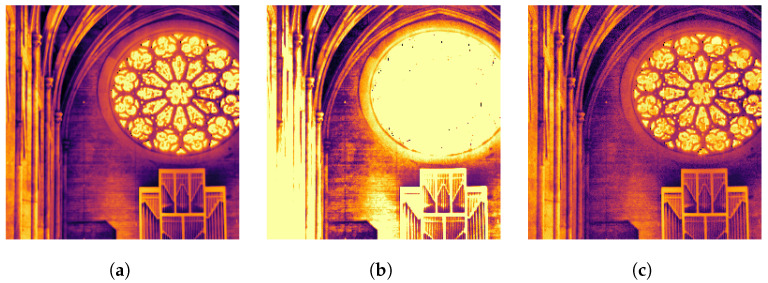
Simulation showing how FAD-SPADs mitigate issues caused by counter saturation. All images are tonemapped using MATLAB’s built-in tonemap function. (**a**) Original HDR image; (**b**) Counter saturation; (**c**) Ours.

Figure 9 simulates a Haar-connected 32 × 32 pixel FAD-SPAD array. Figure 9a is the original linear photon flux in the original scene (from [47]). Here we simulate the passive imaging process, assuming a flux range between 0–10 photons per window and 1000 frames. In Figure 9b,c, a Monte-Carlo simulation generates a probabilistic photon arrival sequence for 1000 time windows. In Figure 9b, we simulate capping the counter at 10 bits, which causes loss of contrast and details at high flux regions as the counters are saturated. In Figure 9c, a 32 × 32 simulated chip is scanned across the scene. The simulated photon arrivals increment and decrement counters connected in a Haar configuration. The image is then reconstructed by solving Equation (2) using MATLAB’s built-in fsolve function, and the next 32 × 32 window is scanned. We again allow only 10 bits of information per counter, but our first-arrival differential approach preserves the entire dynamic range of the scene, even with bit-depth limited counters.

### 4.5. Proof of Concept Prototype in 180 nm CMOS

**Figure 10:**
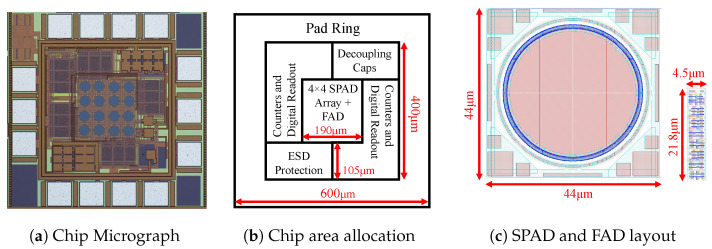
(**a**) Our 16 pixel prototype implemented in 180 nm CMOS. (**b**) Support circuitry such as counters and digital readout are placed outside of the array, with the pixels and FAD circuits in the center. (**c**) Area comparision between a single SPAD pixel (**left**) and a FAD unit (**right**). Figures (**a**,**b**) reproduced with permission from [40].

As a proof of concept, a 16-pixel prototype was fabricated in TSMC’s 180 nm CMOS process (Figure 10). The fabricated chip utilized a Haar-grouped clustering architecture. Only the FAD unit must be placed near the pixels inside of the array; the counters and other digital logic for timing and readout can all be placed outside of the array. We achieve a 36% fill factor with this layout.

**Figure 11:**
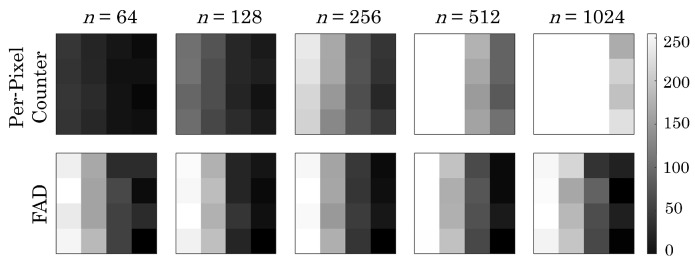
Comparison between the results from our chip and a per-pixel counter when a light gradient is projected onto the chip. The columns show results for different numbers of cycles (*n*). The color scale represents the total counts, and any value over 255 is clipped [40]. Reprinted with permission from [40].

Using the fabricated prototype, we demonstrated that this architecture can achieve background rejection while preserving local gradient information under low and high background conditions. Figure 11 presents a comparison between the FAD-connected chip response versus a per-pixel counter with the same bit depth. A gradient pattern is projected onto the array, and the number of frames (e.g., exposure time) is increased. Where a per-pixel counter will saturate under long exposure, and lose contrast under short exposure, the first-arrival differential structure allows us to preserve the gradient under any exposure conditions. Please refer to [40] for more details.

## 5. The Active Regime: Encoding the First Arrival as Depth Difference

In addition to measuring flux differences, the FAD unit can also indicate the order of arrival of photons originating from a pulsed source, allowing for 3D imaging and range-finding. Such imaging is often performed via Flash LiDAR, which utilizes SPAD arrays to perform single-shot 3D imaging without the need for mechanical scanning [2]. However, such SPAD arrays typically require per-pixel timing circuits called time-to-digital converters (TDCs) with high spatial footprint and data throughput, limiting the spatial and temporal resolution of such systems. The fusion of a few absolute measurements with dense differential measurements allows us to perform single-shot range finding, which we term FAD-LiDAR. Using the FAD technique, we design a flash LiDAR system that can perform high-resolution 3D imaging and scene inference [41]. In this section, we review the working principles, mathematical formulations, and processing algorithms for this architecture. We demonstrate a wide range of 3D inference tasks and depth imaging, as well as study how various realistic factors impact the performance of FAD-LiDAR.

### 5.1. Choice of Architecture

When the FAD unit is configured as in Figure 5, right, it provides local gradients between pixels. Since real images contain discontinuities between pixels, there may be edges in the scene that exceed the bit depth of FAD’s counter, and so we require some sparsely placed TDCs throughout the array to provide anchor points for the depth image. We note that in our approach, these sparsely placed TDCs do not need to be shared by groups of SPADs, as in the shared TDC designs in Figure 1; they are individual measurements at a single point, and this information is used jointly with the FAD local differential information to resolve edges and depth maps. The ratio of the FAD-connected pixels to TDC-connected pixels can be on the order of hundreds; a more more complete anlaysis is given in Section 5.4.3.

### 5.2. Principle and Mathematical Formulation

A FAD unit captures the relative order of photon arrivals at the two pixels. There exists a unique correspondence linking the differential measurement to depth variations between the two pixels.

**Figure 12:**
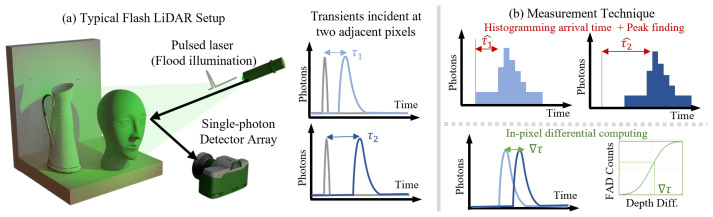
Flash LiDAR typically uses a pulsed light source to flood illuminate the scene and a SPAD array to capture the photon arrival data (**a**). Timing circuits measure the photon arrival profile, and the full histogram of photon arrival information ((**b**), **top**) is reported, and depth is inferred from the difference in the histogram peaks. By measuring this difference directly ((**b**), **bottom**), we reduce the data transmitted and achieve high resolution depth imaging. Reproduced with permission from [41].

Consider a setup as shown in the left section of Figure 12, with the laser and detector collocated. Assume SPAD pixel 1 points to a scene location that is closer to the detector, and SPAD pixel 2 to a farther location. We further assume there was at least one arrival at both SPAD pixel 1 and SPAD pixel 2 during this window. This assumption will be explained in Section 5.2. Then, within a time window, photons reaching SPAD pixel 1 are more likely to arrive earlier than photons from SPAD pixel 2. Across many cycles, the relative frequency of the first photon arrivals between the pixels conveys information about depth difference Δd. This leads to a monotonic mapping between the FAD measurements, FAD, and the depth difference, Δτ, as shown in Figure 12, center. We denote the FAD measurements here as $FAD_{\&}$, as we have imposed the requirement that both SPAD 1 and SPAD 2 received a photon.

(8) $$\begin{array}{c} E[FAD_{\&}] \end{array}\propto -N_{\mathrm{cycles}}\alpha_{1}\alpha_{2}\mathrm{erf}\left(\frac{\Delta \tau }{2\begin{array}{c} \sigma_{T} \end{array}}\right)$$

where $N_{\mathrm{cycles}}$ is the total number of laser cycles and $\alpha_{1}$, $\alpha_{2}$ are the photon flux (per cycle) at the two pixel locations. We model the temporal response of the laser combined with the SPAD jitter as a Gaussian pulse with standard deviation $\sigma_{T}$.

We can acquire intensity estimates $\hat{\alpha_{1}}$, $\hat{\alpha_{2}}$ by using intensity measurements. To decouple illumination effects caused by single photon arrivals, we (1) enable the AND gate so that only when both pixels receive returning photons does FAD perform a comparison (2) measure intensity values at each pixel and factor them out. After these operations, we reach a normalized FAD measurement $nFAD_{\&}$ that is only dependent on the relative depth between two pixels.

(9) $$\begin{array}{cc} \begin{array}{c} nFAD_{\&} \end{array} & =\frac{FAD_{\&}}{N_{\mathrm{cycles}}{\hat{\alpha }}_{1}{\hat{\alpha }}_{2}} \end{array}$$

(10) $$\begin{array}{cc} E[nFAD_{\&}] & \propto -\mathrm{erf}\left(\frac{\Delta \tau }{2\begin{array}{c} \sigma_{T} \end{array}}\right) \end{array}$$

#### Sorting Photon Arrival Types

**Figure 13:**
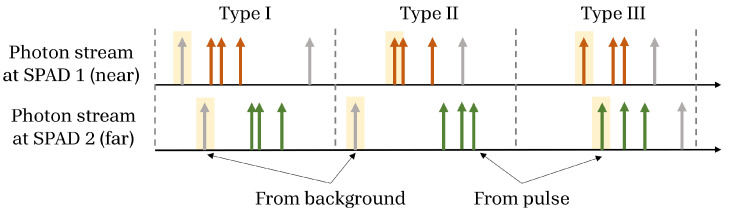
Illustration of possible dual arrival types. Colored arrows (with two colors corresponding to two adjacent SPADs) indicate signal photons, and grey arrows are background photon arrivals. The first arrival on each SPAD may come from signal or background.

Within a cycle, there are three possibilities (see Figure 13): (1) neither SPAD has a photon event (null), (2) only one of the SPADs has a photon event (single), or (3) both SPADs respond to a photon event (dual). Dual events are most likely to be photons reflecting off of nearby points on a surface, and so we are only concerned with these. The hardware is designed not to respond during null cycles. However, single arrivals would cause an increment on the counter. This is a case where the AND gate described in Section 3.1 is employed to reject single-arrival events.

Dual events may come from background (e.g., ambient light or dark counts) or foreground (e.g., reflection of the scene from a laser pulse), with three possible sources:

- Type I. Photons at both SPADs come from the background. Under relatively constant background conditions, these will, on average, cancel out in equal up/down counts.
- Type II. One SPAD receives a photon from the laser pulse, and the other SPAD receives a photon from the background. Under certain conditions, the number of these events are very small relative to the total counts and can be ignored.
- Type III. Both SPADs receive photons from pulses, providing us with differential time of flight data.

**Figure 14:**
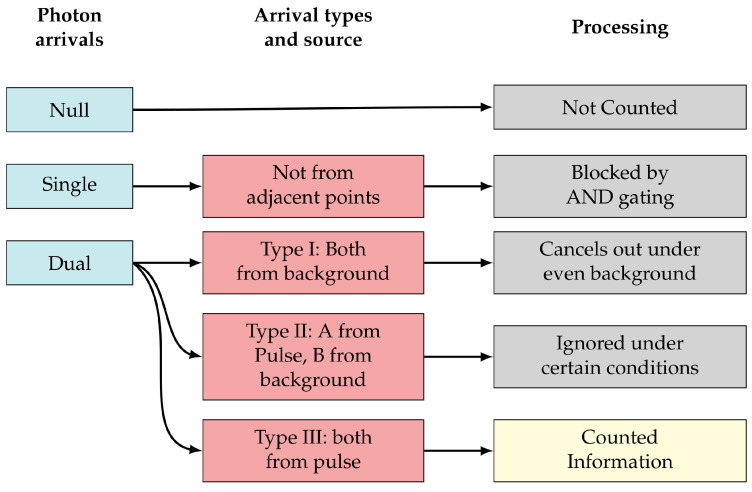
Classification of photon arrivals and how they are processed in our system.

Figure 14 summarizes how photon arrivals are filtered to extract relevant information. Type I and Type II can be ignored under relatively even, low background and over a long-term average. In the short term, the shot noise resulting from the signal and background on two adjacent SPADs does not cancel; however, under our assumption of low background, it will not have a large impact on performance. The shot noise will occupy some of the counter’s bit depth, and this must be taken into consideration when designing the counters, especially under high background conditions. A thorough analysis of the appropriate bit depth for different applications is outside the scope of this paper, but we note heuristically that locally redundant information is rejected FAD unit, allowing more headroom on the counter for shot noise. For more details, please refer to [48].

### 5.3. Edge Inference and Gradient Estimation

The edge can be inferred by applying a thresholding operation on nFAD. We invert the nFAD formula by moment-matching to obtain coarse gradient estimates:

(11) $$\hat{\Delta \tau }=\begin{array}{c} -2\sigma_{T}\mathrm{inverf}\left(nFAD_{\&}\right) \end{array}$$

Here, inverf corresponds to the inverse error response function. From the depth gradients in both *x* and *y*, an initial per-pixel surface normal map approximation can be obtained [49]. The normal vector n for each pixel can be derived as:

(12) $$n_{u}={\left[{\hat{\partial }}_{x}z,{\hat{\partial }}_{y}z,-1\right]}^{t},n=\frac{n_{u}}{\left|\right|n_{u}\left|\right|}$$

We also provide corrections to these formulas under the presence of some background ambient light in Section 4 in [48].

Here we do not use group-connected SPAD pixels, but rather connect each SPAD to its four cardinal nearest neighbors via FAD unit in order to obtain local gradients corresponding to surface normals in the scene. Such a configuration allows us to capture information such as edges, surface gradients, and normals, for which relative or differential arrival is sufficient. In contrast to the passive case, we require an active lighting source (e.g., a pulsed laser). In the case of differential sensing applications (edges, gradients), FAD does not require the laser to be synchronized with the SPAD timing windows. Such a relaxation simplifies our hardware connectivity greatly as compared to typical TDC-based approaches.

**Figure 15:**
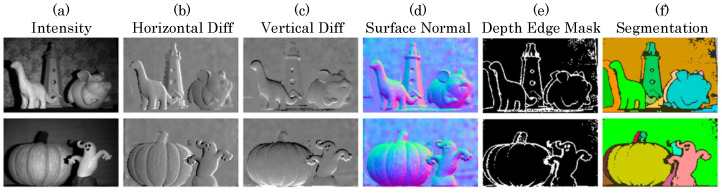
3D imaging applications of FAD LiDAR. Column (**a**): intensity view of the scenes. Columns (**b**–**f**) correspond to different 3D applications as labeled in the figure. This is a partial reprint, used with permission, of a figure from [41].

Using FAD units and nearest-neighbor connectivity, we directly perform 3D scene inference tasks as shown in (Figure 15). Tasks such as depth edge detection, depth-based segmentation, and normal estimation are sufficient with FAD measurements (relative depth information) and per-pixel intensity estimates. Depth edge detection and segmentation can be performed by appropriately thresholding nFAD measurements as above, while normal estimation requires a two-step procedure: first performing non-local denoising of the raw FAD measurement, inverting relative depth difference from nFAD, then performing Poisson integration [50] to generate clean surface normal estimates.

#### Depth Reconstruction Using FAD-LiDAR

For the purpose of 3D imaging or depth reconstruction, we must therefore include some TDCs to anchor points in the image to absolute values (but far fewer than one TDC per pixel). There are two main reasons that a few sparse TDCs are needed: (1) the sparse TDCs provide absolute depth references, and (2) at a large depth gap, the scene point closer to the sensor will always result in photon arrivals earlier than the further point. In other words, FAD counters saturate. Sparse TDCs can help resolve the information loss at the gap. In Section 5.4.3, we study how varying the TDC connection density affects the final depth reconstruction.

**Figure 16:**
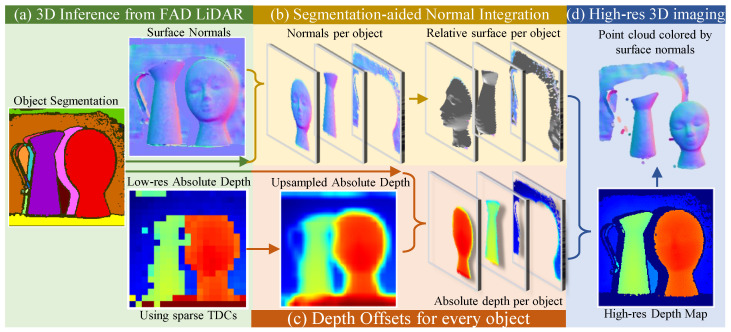
FAD LiDAR 3D imaging pipeline. FAD units enable the estimation of surface normals and object segmentation (**a**). Aided by sparse depth from TDCs, we demonstrate high-resolution 3D imaging (**d**). Using the object segmentation, we integrate the surface normals per object to get the relative surface (**b**) and apply offset per object using the upsampled absolute depth (**c**).

**Figure 17:**
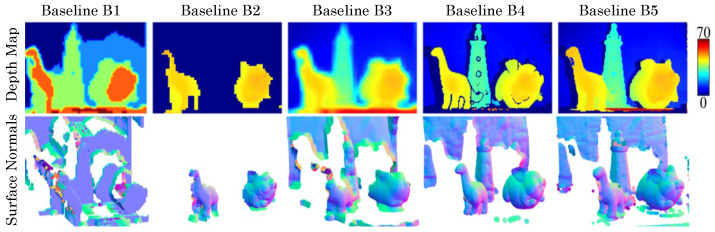
High-resolution 3D imaging and surface normals with emulated FAD LiDAR. FAD LiDAR implemented on a single-pixel SPAD system enabled high-quality 3D reconstruction and surface normal estimation comparable to scanning LiDAR ground truth. In contrast, conventional flash LiDAR designs B1, B2, and B3 (ref. [41]) exhibit performance tradeoffs, resulting in poor depth resolution (B1), range (B2), or spatial resolution (B3). Our differential flash approach provides significantly improved reconstruction quality relative to conventional baselines at matched data throughput. This is a partial reprint, used with permission, of a figure from [41].

Our depth processing pipeline is divided into three main blocks, as shown in Figure 16. First, we perform non-local means denoising to the raw nFAD measurements and perform edge detection. Then we apply binary morphological operations to segment discontinuous objects. We extract surface normals for each segmented object and obtain the relative surface per object, as shown Figure 16b. Then we align the relative surfaces with low-resolution absolute depth captured by sparse TDCs. This procedure allows us to obtain a high-resolution depth map across the entire array. In column Figure 17, column 4, we show high-quality depth reconstruction results using our approach. We demonstrate via emulation that FAD-LiDAR provides improved performance for the same data bandwidth (Figure 17). In these baselines, either spatial, temporal resolution, or range is sacrificed to achieve the same data bandwidth.

### 5.4. Performance Characterizations

In this section, we study how different factors impact the performance of FAD-LiDAR. By simulating between a pair of pixels, we study the effect of albedo variation, the presence of background, and how the device jitter impacts the depth resolution. We also study how TDC density and exposures impact the FAD-LiDAR depth reconstruction performance as a whole.

#### 5.4.1. Effects of Albedo Variation and Background

**Figure 18:**
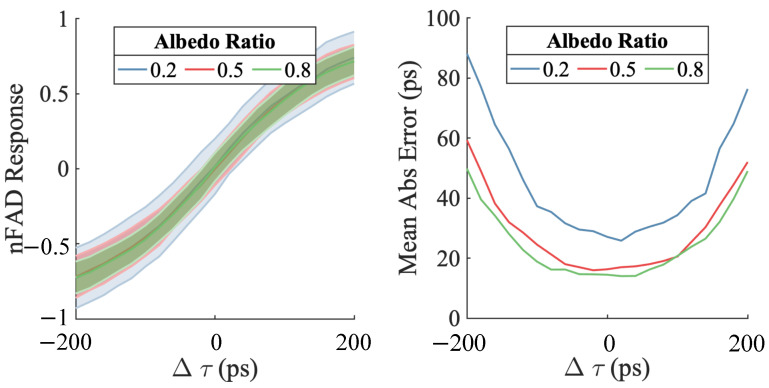
Examining albedo variation effects under low ambient light (dark count rate of 1000 cps). With fixed $\alpha_{1}$ and varying albedo ratio $\alpha_{2}/\alpha_{1}$, increasing albedo ratio leads to greater variance in nFAD and depth inversion error due to fewer dual arrivals. Under 1000 cps dark count conditions, altering albedo introduces no systematic bias owing to the low background. Reproduced with permission from [41].

To examine the impacts of changes in albedo and background noise, we conduct simulations on a pixel pair. The pulse intensity of SPAD 1 was held constant at 0.01. Three albedo ratios were tested: 0.3, 0.5, and 0.8. We consider no background illumination in this simulation and only dark counts were present. The outcomes of varying the albedo ratio are presented in Figure 18.

**Figure 19:**
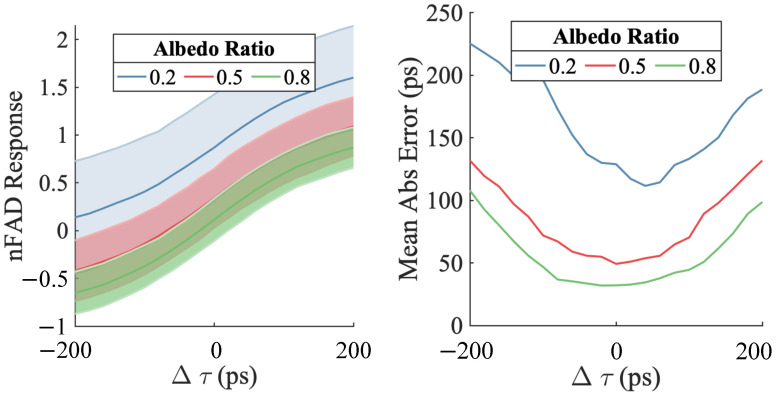
Effect of albedo variation under high ambient light. The left side demonstrates that with both high ambient light and significant albedo differences, the nFAD measurements become biased. At Δτ=0, the expected value of nFAD measurement won’t converge to zero. The right side shows depth gradient estimation errors growing as the albedo ratio increases. Notably, errors become very large and asymmetric when the ratio exceeds 0.5. Thus for pairwise estimation, the method is effective for albedo ratios up to 0.5.

Figure 19 demonstrates the impact of increasing albedo variation under the presence of significant background. The left side of the figure indicates that as albedo variation increases, the bias in the nFAD measurement without proper correction also increases. The right side shows that depth estimation error also rises with increasing albedo ratio as a consequence of fewer dual arrival events.

**Figure 20:**
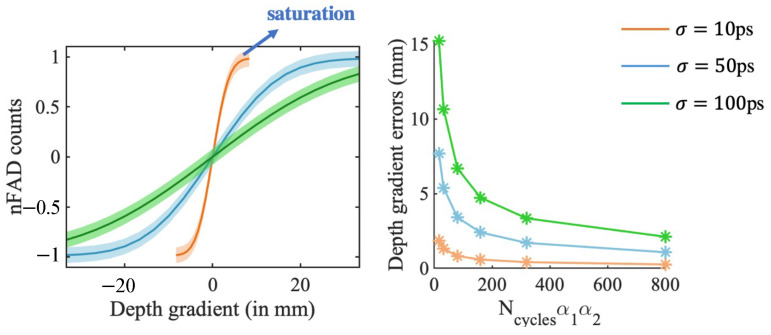
Effect of jitter on range and resolution. (**left**) nFAD response for 3 different jitter values. (**right**) resolution as a function of average dual arrivals for 3 jitter values.

Figure 20 illustrates how increasing jitter values of 10 ps, 50 ps, and 100 ps impact the detectable gradient range and depth resolution. Larger jitter enables detection of a wider range of gradients, but reduces depth resolution. This is because higher jitter incorporates greater pulse width, resulting in more uncertain photon arrival times and increased depth errors. Smaller jitter provides finer resolution, albeit over a shorter range, since less overlap is needed between two narrower signals for clear edge detection. At the extreme end, if the transients were dirac delta signals with perfect temporal precision, any FAD detection would simply indicate depth gradient direction without any information on the value of the depth gradient.

#### 5.4.2. Effect of Exposure

**Figure 21:**
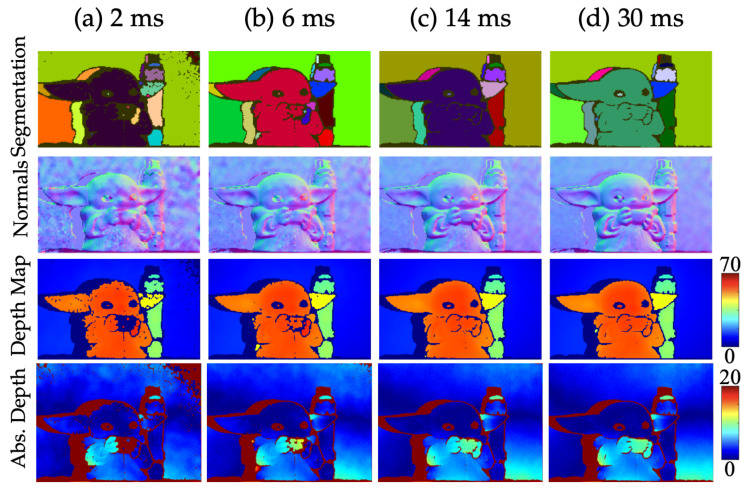
Impact of exposure time on 3D imaging. Even with reduced $T_{\mathrm{int}}$, FAD-LiDAR still achieves successful object segmentation, approximation of surface normals, and depth mapping. The color bars for the depth maps and the error maps are in units of cm.

To examine the effects of exposure on 3D imaging capabilities, we systematically varied exposure time (effectively modifying the number of dual arrivals) and evaluated performance at estimating object segmentation, surface normals, and depth maps as presented in Figure 21. We also plotted the absolute error between our estimated depth map and the known ground truth depth map. Our approach performed well even at 6ms exposures for both segmentation and surface normals. However, we observed degraded quality in surface normal and depth reconstruction as exposure decreased.

#### 5.4.3. Effect of TDC Sparsity

**Figure 22:**
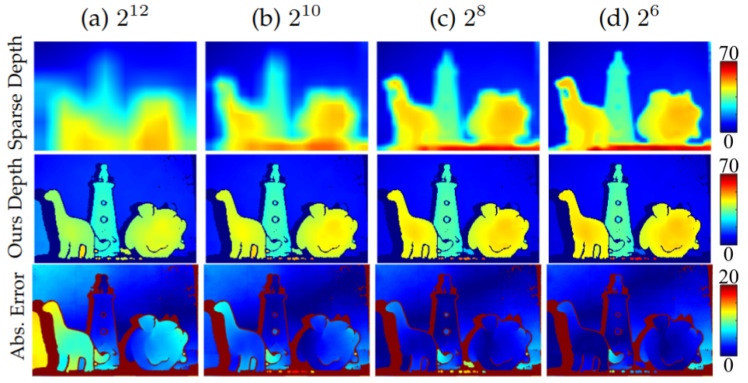
The values at the top of the columns are the number of SPADs per TDC. The top row illustrates only the information collected by the TDCs, and the middle row includes the differential SPAD information. While high-frequency information is maintained in each case due to the differential nature of the FAD unit, the absolute error in the depth estimation decreases as more TDCs are added. In column a, for example, with $2^{12}$ SPADs per TDC, we get low accuracy. As we add more TDCs in columns b–d, the accuracy of the true depth information increases, at the cost of a higher bandwidth.

As described in Section 5.1, some absolute timing (depth) information is required via TDCs. Figure 22 demonstrates the effect of varying the ratio of FAD-SPAD pixels to TDC-connected SPADs on performance. The SPAD resolution is fixed to 512×384 and number of TDCs is varied. The first row shows the depth interpolated from sparse TDC measurements. The second row shows our depth reconstruction using our segmentation-aided reconstruction. The last row shows the absolute errors as compared to a high-resolution scan. With more TDCs, the absolute depth measurement has better resolution at the cost of higher spatial footprint and throughput. Our approach fuses sparse absolute information with high resolution relative depth information.

## 6. Discussion

Having illustrated the functioning of a FAD unit, and demonstrated two applications for FAD-SPAD architecture, we now turn to a discussion of other possibilities and design considerations for the use of FAD circuits in SPAD arrays.

### 6.1. Array Connectivity

**Figure 23:**
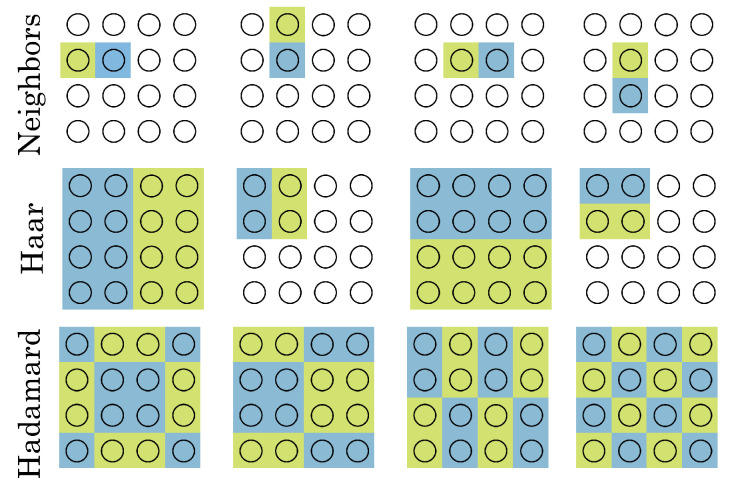
Possible connectivity schemes for clusters of SPADs. Each block of 16 circles (SPADs) represents one FAD unit’s connections, and green and blue correspond to “up” or “down” counts. At the top, in the nearest-neighbors architecture, each set of nearest neighbors has a FAD unit between. In the Haar arrangement, clusters of many SPADs are grouped together.

Some possible means of connecting the SPADs in an array via OR gates and FAD units are shown in Figure 23. In this work, we used nearest neighbors (top row) for depth sensing and Haar (second row) for HDR imaging, but any other grouping is possible, e.g., a Hadamard-transform grouping, as in the third row, or even random clusters. In any connection scheme where multiple pixels feed into the same FAD circuit, it is critical to match the path lengths of the traces from the SPAD output to the OR gates and SR latch input, and carefully control propagation delays. For that reason, the simplest of these approaches to scale to larger arrays is nearest neighbors.

The optimal choice of connectivity, as well as whether or not to permit single/dual arrivals, depends on the application.

### 6.2. Pixel Response Non-Uniformity (PRNU) and Fixed-Pattern Noise Correction

Individual SPADs have varying offsets (dark counts) and gain (responsivity) due to unavoidable variations in doping that occur during fabrication. With independently operating SPADs, one can simply measure the array’s response to no light and subtract this offset, and calibrate each pixel individually for its gain.

While the gains and offset will be consistent and roughly linear for each SPAD within a certain range of incident flux, the differences between the responses of the pixels are not linear. That is, the bias we observe between two pixels or groups depends not only on the individual sensitivity of the SPADS, but also on the illumination. This is especially true in grouped architectures, such as the Haar arrangement in Section 4.1. The Haar-like architecture means that a single “hot” pixel dominates the behavior of the counts in its local block as well as every block higher up in the hierarchy.

However, it is still possible to recover and calibrate for these biases by setting up an appropriate quadratic minimization problem. We begin with the assumption that each SPAD in the array has a unique and consistent gain term that describes its sensitivity to light flux, and express that as *P*, where each $p_{ij}$ is a Gaussian variable with mean μ=1. The variance may not be known *a priori*, but it can be discovered experimentally. The recorded output *r* is the flux times this sensitivity, multiplied element-wise:

(13) r=P∘Φ

Capturing a real-world image with a small array requires scanning it across a scene, so we will have many matrices *r*, which are assembled into a matrix *R* by taking each *r* as a row vector. The measured flux is then the measurements time the reciprocal of each of the sensitivities:

(14) $$\Phi =R\circ P^{-1}$$

While this is a highly over-determined system, natural images are typically sparse in local gradients of flux. We also construct a series of matrices $g_{ij}$ to penalize local spatial gradients. *G* is a concatenation of these flattened matrices. Then we would like to minimize over ΦG, with a constraint that *P* is bounded to some tolerance *t*:

(15) $$\min_{P}{\left|\left(R\circ P^{-1}\right)G\right|}_{2}^{2}$$

(16) $$\begin{array}{cc} s.t.\; & 1-t<P<1-t \end{array}$$

Then, given *P*, we can calibrate the FAD readout measurements before performing reconstruction.

## 7. Summary and Discussion

In this paper we present techniques and supporting analysis for a novel type of SPAD array design based on differential sensing. We also present two applications: HDR imaging and 3D imaging, featuring two architectures (Haar and nearest neighbor). However, this is only a sample of the capabilities enabled by FAD architectures.

The differential nature of the nearest-neighbor FAD-SPAD approach lends itself naturally to contrast-enhancement and edge detection. For example, the differential nature of FAD units inherently amplifies local differences and thus could enhance contrast in bioimaging applications, such as the loss in contrast due to scattering. On the other hand, a clustered grouping, such as the Haar version, produces hierarchical data. This can be useful for event-based cameras, where we may only be interested in smaller regions of rapid change within a scene. It may also be useful for further on-chip compression techniques, where less information may be reported for flat regions of the image that contain little differential information, and more bits may be used to preserve high-frequency information in other regions.

There is also fertile space for analysis of other differential connectivity schemes to enable compressed sensing of images. Binary compressed sensing matrices (e.g., Hadamard transforms) can be implemented similarly to our Haar example for HDR by simply changing the connected groups. The differential grouped measurements could also be used to do adaptive sensing in sparse image acquisition. For example, if a large differential signal is found in one region of an image, then a smart sensor could continue to collect finer-grained measurements in that region, and not collect redundant data in a region of the image that lacks contrast.

Finally, the gains in circuit footprint and scalability of the concept we show here could facilitate the development of larger and denser SPAD arrays with high photon detection probability. We hope this work will inspire further development of unconventional SPAD array designs.

## Figures and Tables

**Table 1:** A summary of the two applications demonstrated in this paper. Under passive lighting conditions, we consider the FAD-SPAD to replace a photon-counting method, and show how we are able to discern a high dynamic range of fluxes. Under active lighting with a pulsed laser, the FAD-SPAD replaces a TDC, and relative timing is used for flash LiDAR.

Application	HDR	LiDAR
Δ	flux	time of arrival
Function	photon-counting	photon-timing
Lighting	passive	active
TDCs	none	few

## Data Availability

No new data were created or analyzed in this study. Data sharing is not applicable to this article.
